# RCAN-11R peptide provides immunosuppression for fully mismatched islet allografts in mice

**DOI:** 10.1038/s41598-017-02934-3

**Published:** 2017-06-08

**Authors:** Hirofumi Noguchi, Koji Sugimoto, Chika Miyagi-Shiohira, Yoshiki Nakashima, Naoya Kobayashi, Issei Saitoh, Masami Watanabe, Yasufumi Noguchi

**Affiliations:** 1 Department of Regenerative Medicine, Graduate School of Medicine, University of the Ryukyus, Okinawa, 903-0215 Japan; 2 Department of Surgery, Yoshinogawa Medical Center, Tokushima, 776-8511 Japan; 3 Okayama Saidaiji Hospital, Okayama, 704-8192 Japan; 4 Division of Pediatric Dentistry, Graduate School of Medical and Dental Science, Niigata University, Niigata, 951-8514 Japan; 5 Department of Urology, Okayama University Graduate School of Medicine, Dentistry and Pharmaceutical Sciences, Okayama, 700-8558 Japan; 6 Department of Socio-environmental Design, Hiroshima International University, Hiroshima, 737-0112 Japan

## Abstract

Calcineurin inhibitors have been used for transplant therapy. However, the inhibition of calcineurin outside the immune system has a number of side effects. We previously developed a cell-permeable inhibitor of NFAT (nuclear factor of activated T cells) using the polyarginine peptide delivery system. This peptide (11R-VIVIT) selectively interferes with calcineurin-NFAT interaction without affecting the activity of calcineurin phosphatase and provides immunosuppression for fully mismatched islet allografts in mice. However, our recent study showed that 11R-VIVIT affected cell viability *in vitro* when it was used at higher concentration because of the VIVIT sequence. The aim of this study is to develop a safer NFAT inhibitor (RCAN-11R) that does not affect cell viability, and which is less toxic than calcineurin inhibitors. The minimal sequence of the protein family of regulators of calcineurin (RCAN) that is responsible for the inhibition of calcineurin-NFAT signaling was recently characterized. The peptide could selectively interfere with the calcineurin-NFAT interaction without affecting the activity of calcineurin phosphatase, similar to 11R-VIVIT. RCAN-11R did not affect cell viability when it was used at a higher concentration than the toxic concentration of 11R-VIVIT. RCAN-11R could therefore be useful as a therapeutic agent that is less toxic than current drugs or 11R-VIVIT.

## Introduction

An important mechanism whereby calcineurin promotes the activation of T cells and the induction of cytokine-related genes is largely attributed to a family of transcriptional regulators that are referred to as NFAT. The immunosuppressants cyclosporine A and FK506, which are used clinically to prevent transplant rejection, inhibit the activity of calcineurin phosphatase on all its protein substrates, including NFAT^1, 2^. Although these drugs have revolutionized transplant therapy, their use is associated with the progressive loss of the renal function, hypertension, hyperglycemia, neurotoxicity and an increased risk of malignancy^3–6^. We previously developed a cell-permeable NFAT inhibitor using the polyarginine peptide delivery system^7^. An NFAT inhibitor peptide, VIVIT, was developed based on the conserved calcineurin docking site of the NFAT family^8^. The peptide selectively interferes with the calcineurin-NFAT interaction without affecting the calcineurin phosphatase activity, suggesting that it is useful as a therapeutic agent that is less toxic than current drugs. We synthesized VIVIT peptide as a C-terminal fusion protein with 11-arginine (11R) to facilitate the efficient *in vivo* delivery of the NFAT inhibitor peptide into T cells. Protein transduction domains such as polyarginine have been developed to deliver bioactive peptides and proteins into eukaryotic cells^9–13^ and to successfully deliver covalently attached peptides and proteins *in vivo* into all mamalian tissues^14^. 11R-VIVIT transduced lymphocytes, and specifically inhibited NFAT reporter activity to a significant extent. Moreover, the peptide provided immunosuppression for fully mismatched islet allografts in mice. In addition, it did not affect the secretion of insulin, whereas FK506 caused a dose-dependent decrease in the secretion of insulin. Thus, the peptide had been expected to have clinical applications because it was less toxic than calcineurin inhibitors^7^. However, our recent study showed that at a concentration of >10 μM, 11R-VIVIT had unexpected effects on cell viability. In a detailed study, we concluded that this was due to the VIVIT sequence, not the 11R sequence.

Recently, the minimal sequence of the protein family of regulators of calcineurin (RCAN, previously known as calcipressins or DSCR1)^15^, which is responsible for the inhibition of calcineurin-NFAT signaling, was characterized^16^. RCAN physically interacts with calcineurin and modulates its phosphatase activity^17–20^. The RCAN-derived peptide spanning this sequence binds to calcineurin with high affinity. A peptide spanning the NFAT PxIxIT sequence, which binds to calcineurin and facilitates the dephosphorylation and activation of NFAT, competes with this interaction. Similarly to the VIVIT peptide, the RCAN-derived peptide does not inhibit the general calcineurin phosphatase activity, suggesting that it may have a specific immunosuppressive effect on the calcineurin-NFAT signaling pathway (Supplemental Fig. 1).

In the present study, we developed another safer NFAT inhibitor (RCAN-11R) which was less toxic than calcineurin inhibitors or 11R-VIVIT. We synthesized the RCAN peptide as an N-terminal fusion protein with 11-arginine (11R) in order to achieve the efficient *in vivo* delivery of the RCAN peptide into T cells.

## Results

### The delivery of RCAN-11R peptide into T cells

**Figure 1:**
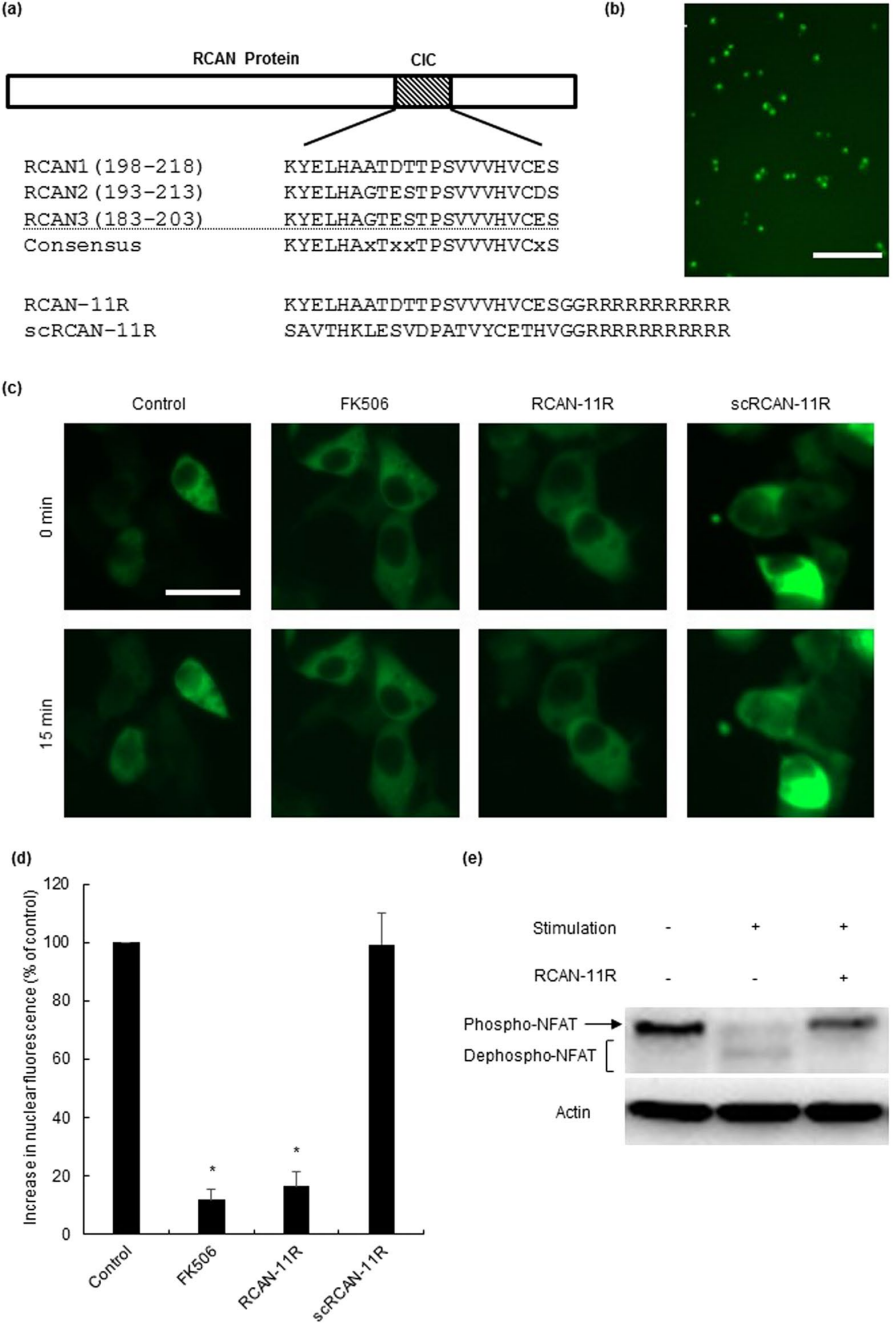
The transduction of RCAN-11R into lymphocytes and the inhibition of NFAT nuclear translocation. (**a**) The CIC (Calcineurin inhibitor of Calcipressin) motif of the human RCAN protein family (upper schematic illustration) and the sequence of RCAN-11R and the scramble RCAN-11R peptides (lower schematic illustration). (**b**) Transduction of RCAN-11R into lymphocytes. Jurkat cells were incubated with 10 μM FITC-RCAN-11R and examined using an Olympus confocal microscope. Scale bar = 100 µm. (**c**) The inhibition of NFAT nuclear translocation. HEK 293 cells that had been transfected with GFP-NFAT-1 plasmid were incubated with 1 μM FK506 or 20 μM RCAN-11R/scRCAN-11R for 2 h and then 500 nM ionomycin was added to the culture medium. Scale bar = 20 µm. (**d**) The percentage of NFAT nuclear translocation. GFP-NFAT-1 nuclear translocation was quantified by microscopy. *p < 0.01 in comparison to control (no treatment). The values represent the mean ± SE of five independent experiments. (**e**) Western blot of phosphorylated NFAT1 (Phospho-NFAT) and the faster-migrating dephosphorylated forms (Dephospho-NFAT).

We synthesized RCAN peptide as a N-terminal fusion protein with 11-arginine (11R) to facilitate the efficient *in vivo* delivery of RCAN-11R peptide into T cells. Polyarginine facilitates the uptake of peptides and protein into mammalian cells with high efficiency^9–11^. We also constructed a negative control peptide conjugate (scRCAN-11R) by scrambling the sequence of RCAN amino acids (Fig. 1a). To examine whether RCAN-11R transduces lymphocytes, Jurkat cells were treated with FITC-conjugated RCAN-11R. One hour after transduction, FITC-conjugated RCAN-11R was observed as a fluorescent signal in all of the living Jurkat cells (Fig. 1b, Supplemental Fig. 2).

### The inhibitory effect of the translocation of NFAT by the RCAN-11R peptide

To determine the effect of RCAN-11R on NFAT, we examined NFAT nuclear translocation and NFAT-dependent reporter activity. The inhibition of NFAT nuclear translocation was investigated in 293 cells that were transfected with GFP-NFAT-1^6^. The GFP-NFAT-1 signal was diffusely distributed in the cytoplasm and was absent from the nucleus (Fig. 1c; 0 min). After 15 min of stimulation with 500 nM ionomycin in the medium, alone or with 20 μM scRCAN-11R (control peptide), the fluorescent GFP-NFAT-1 translocated to the nucleus (Fig. 1c; Control 15 min, scRCAN-11R 15 min). In contrast, the presence of 20 μM RCAN-11R blocked the ionomycin-induced translocation of GFP-NFAT-1 from the cytoplasm to the nucleus (Fig. 1c; RCAN-11R 15 min, Fig. 1d). The strong inhibition of GFP-NFAT-1 nuclear localization was also observed with 1 μM FK506 (Fig. 1c; FK506 15 min, Fig. 1d).

To evaluate the ability of RCAN-11R to inhibit NFAT activation, Jurkat cells were stimulated with ionomycin and phorbol 12-myristate 13-acetate (PMA) in the presence or absence of 10 μM RCAN-11R. Stimulation of the cells with 300 nM ionomycin and 20 nM PMA caused dephosphorylation of NFAT. Pretreatment with RCAN-11R resulted in the inhibition of NFAT dephosphorylation (Fig. 1e).

### The inhibition of calcineurin-NFAT pathway by the RCAN-11R peptide

**Figure 2:**
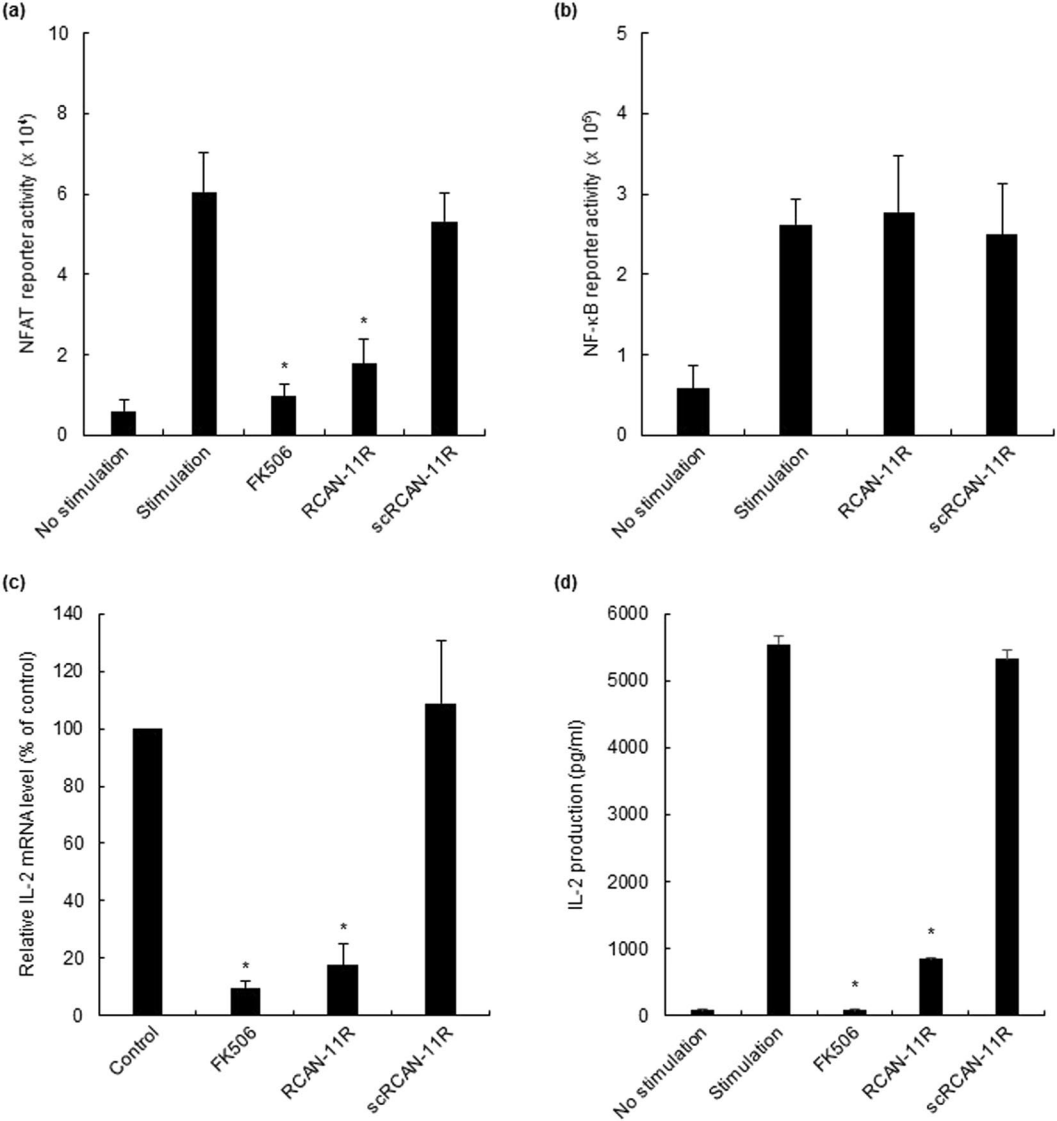
The inhibition of NFAT reporter activity and the production of IL2. (**a**,**b**) NFAT (**a**) and NF-κB (**b**) reporter activity. Jurkat cells were electroporated with 5 μg of pNFAT-SEAP or pNF-κB-SEAP, respectively. The cells were incubated with 1 μM FK506 or 20 μM RCAN-11R/scRCAN-11R for 1 h, and stimulated with 200 nM PMA and 4 μM ionomycin for 12 h. (**c**) The inhibition of IL-2 transcription. Jurkat cells were treated with 1 μM FK506 or 20 μM RCAN-11R/scRCAN-11R for 1 h, then with 200 nM PMA and 4 μM ionomycin for an additional 12 h. The cells were subjected to a quantitative RT-PCR. (**d**) The inhibition of IL-2 production. Jurkat cells were treated with 1 μM FK506 or 20 μM RCAN-11R/scRCAN-11R for 1 h and then incubated with 200 nM PMA and 4 μM ionomycin for an additional 12 h. *p < 0.01 in comparison to stimulation (control) without treatment of FK506 or peptides. The values represent the mean ± SE of five independent experiments.

To examine directly that RCAN-11R specifically inhibits the calcineurin-NFAT pathway, the NFAT-1- and NF-κB-dependent transcription activity in Jurkat cells were examined. We transfected Jurkat cells with NFAT and NF-κB reporter plasmids, then treated the cells with 20 μM RCAN-11R or scRCAN-11R for 1 h and with phorbol 12-myristate 13-acetate (PMA) and ionomycin for an additional 12 h. RCAN-11R specifically and significantly inhibited the NFAT reporter activity (Fig. 2a), but did not inhibit NF-κB-dependent activity (Fig. 2b).

### The effect of RCAN-11R on the transcription and the production of interleukin-2

To test the effect of RCAN-11R on the transcription and the production of interleukin-2 (IL-2), we treated Jurkat cells with 20 μM RCAN-11R, scRCAN-11R or FK506 for 1 h, then with PMA and ionomycin for an additional 12 h. We performed a quantitative RT-PCR to determine the quantity of IL-2 mRNA and analyzed the supernatants using an ELISA kit to determine the quantity of IL-2. The IL-2 transcription in cells treated with 20 μM RCAN-11R was significantly decreased to less than 20% of the level in untreated cells or scRCAN-11R treated cells (Fig. 2c). The level of IL-2 in the supernatant of cells treated with 20 μM RCAN-11R was significantly decreased by 15% in comparison to the untreated cells. The scrambled peptide did not change the protein level; 100 nM FK506 completely inhibited the production of IL-2 (Fig. 2d).

### The dose-response and half-life of RCAN-11R

**Figure 3:**
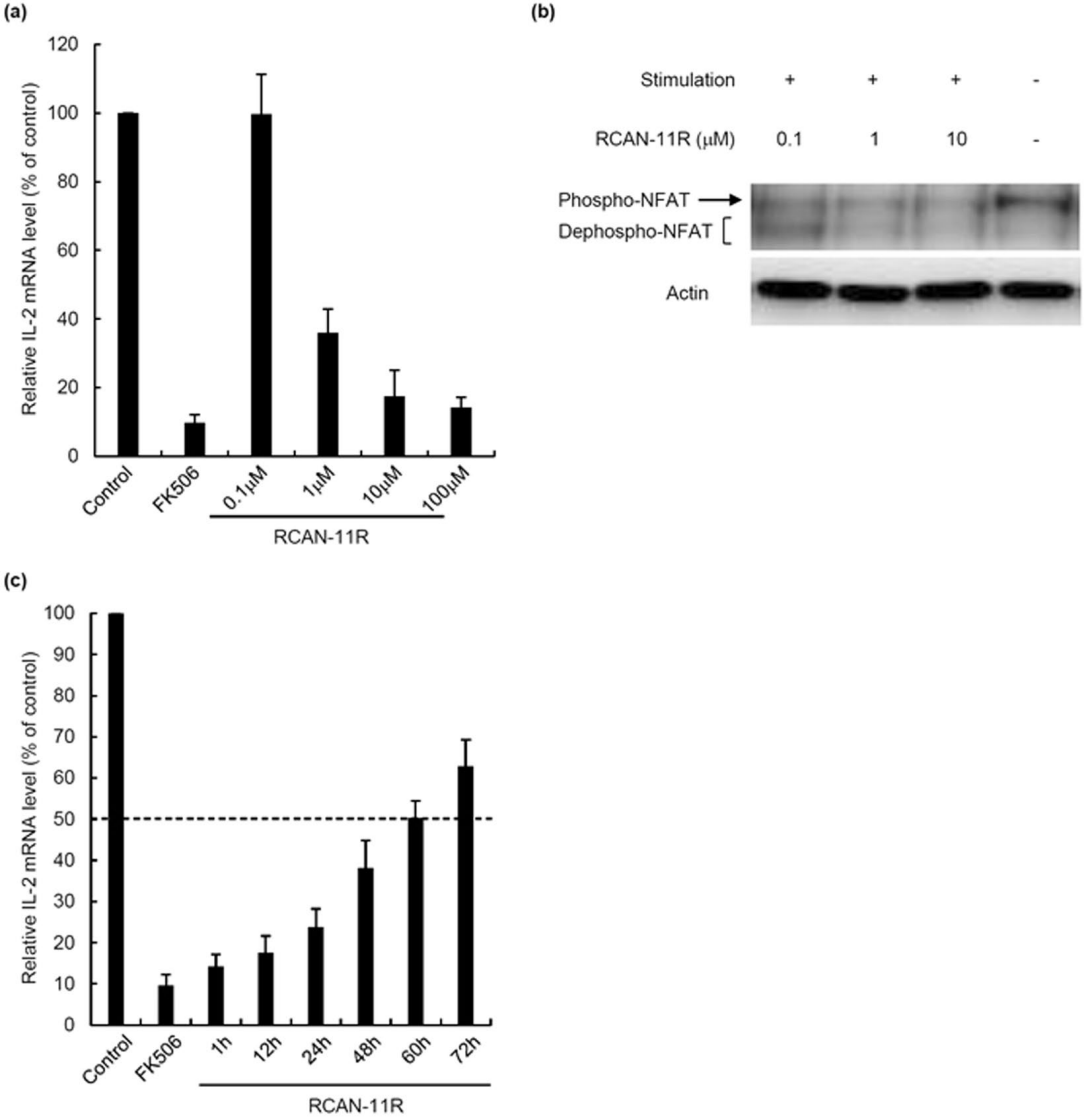
The dose-response and half-life of RCAN-11R. (**a**) The dose-response of RCAN-11R. Jurkat cells were treated with 0.1–100 μM RCAN-11R or 1 μM FK506 for 1 h, then with 200 nM PMA and 4 μM ionomycin for an additional 12 h. The cells were subjected to a quantitative RT-PCR. (**b**) Dephosphorylation of NFAT. Jurkat cells were treated with 0.1, 1, or 10 μM RCAN-11R for 1 h and with 20 nM PMA and 300 nM ionomycin for an additional 15 min and then western blot was performed. (**c**) The half-life of RCAN-11R. Jurkat cells were treated with 20 μM RCAN-11R for periods of 1–72 h or with 1 μM FK506 for 1 h, then with 200 nM PMA and 4 μM ionomycin for an additional 12 h. The cells were then subjected to a quantitative RT-PCR. The values represent the mean ± SE of five independent experiments.

To test the dose-response of RCAN-11R, we treated Jurkat cells with 0.1–100 μM RCAN-11R for 1 h, then with PMA and ionomycin for an additional 12 h. We performed a quantitative RT-PCR to determine the quantity of IL-2 mRNA. The IL-2 transcription in cells treated with RCAN-11R was decreased in a dose-dependent manner (Fig. 3a). To test the dose-response of RCAN-11R on dephosphorylation of NFAT, we treated Jurkat cells with 0.1–10 μM RCAN-11R for 1 h, then with PMA and ionomycin for an additional 15 min. Dephosphorylation of NFAT in cells treated with RCAN-11R was inhibited in a dose-dependent manner (Fig. 3b).

To evaluate the half-life of RCAN-11R in T cells, Jurkat cells were treated with 20 μM RCAN-11R for periods of 1–72 h, then with PMA and ionomycin for an additional 12 h. The cells were then subjected to a quantitative RT-PCR. The functional half-life of RCAN-11R was ~60 h (Fig. 3c).

### The effect of RCAN-11R as an immunosuppressive agent

**Figure 4:**
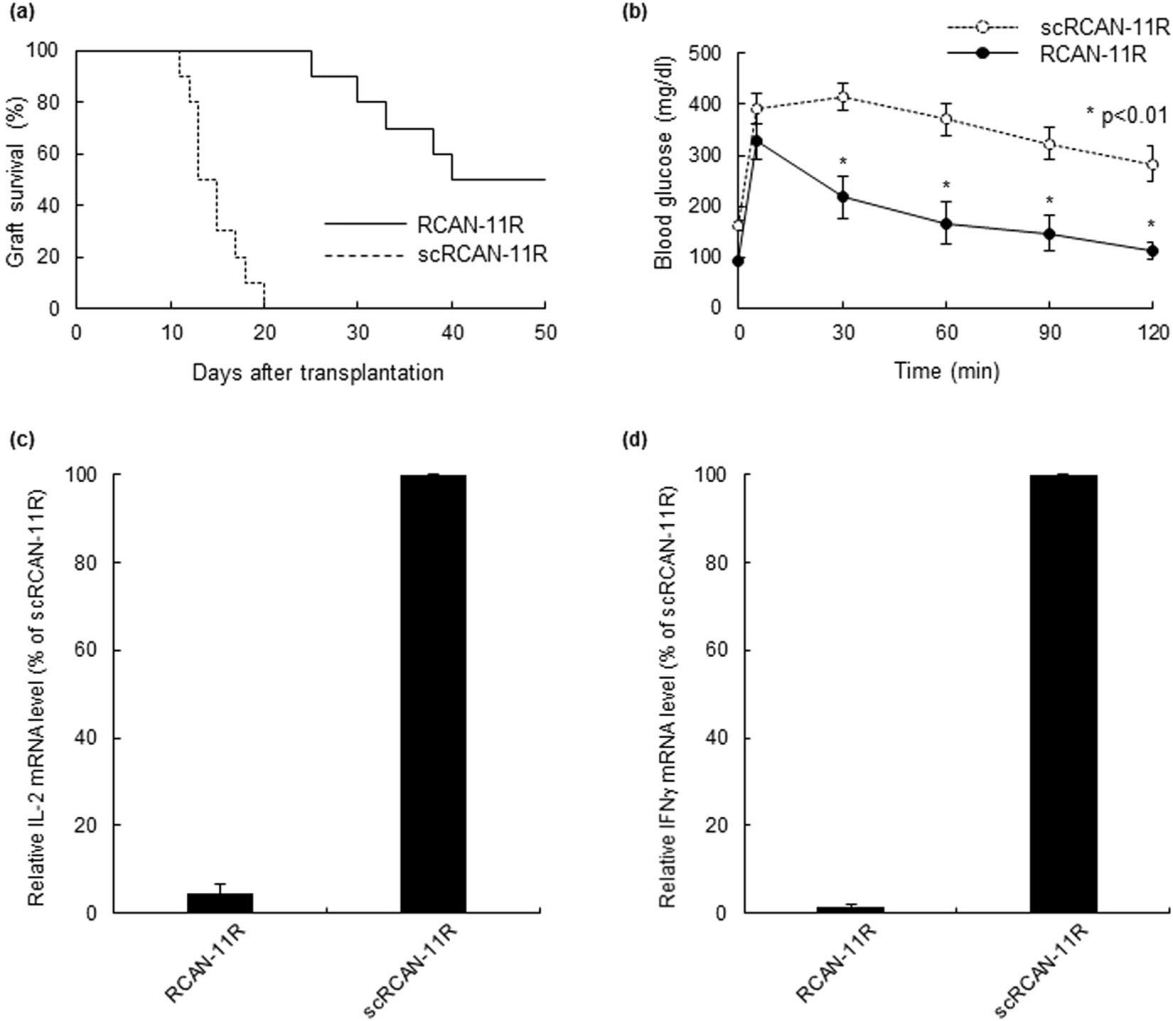
The effects of RCAN-11R on islet transplantation. (**a**) The differences in the duration of graft survival between the RCAN-11R group (n = 10) and the scRCAN-11R group (n = 10), as evaluated by a Kaplan-Meier log-rank test. (**b**) Intraperitoneal glucose tolerance testing (IPGTT). IPGTT was performed ten days after transplantation. Glucose (2.0 g/kg body weight) was intraperitoneally injected. (**c**,**d**) The inhibition of IL-2 (**c**) or IFNγ (**d**) transcription *in vivo*. Ten days after transplantation, islet allografts with lymphocyte (n = 3 each) were surgically obtained from kidneys and the cells were subjected to a quantitative RT-PCR. *p < 0.01 in comparison to scRCAN-11R at the same time. The values represent the mean ± SE.

We investigated the effectiveness of RCAN-11R as an immunosuppressive agent in islet transplantation. C57BL/6 mice were used as donors and BALB/c mice were used as recipients. The BALB/c mice were intraperitoneally injected with 50 mg/kg of RCAN-11R or scRCAN-11R, once per day throughout the study. The mice treated with RCAN-11R had significantly prolonged graft survival (Fig. 4a) in comparison to the scRCAN-11R-treated BALB/c recipients (P < 0.01). RCAN-11R provided immunosuppression for the fully mismatched islet grafts.

Intraperitoneal glucose tolerance testing (IPGTT) was carried out on both groups ten days after transplantation (before rejection). The mice were fasted overnight, after which glucose (2.0 g/kg body weight) was injected intraperitoneally. The blood glucose levels were measured before injection and at 5, 30, 60, 90, and 120 minutes after injection. The blood glucose levels of the mice that were treated with RCAN-11R were significantly lower than those of the mice that were treated with scRCAN-11R at 30, 60, 90, and 120 minutes after injection (p < 0.01) (Fig. 4b).

We investigated cytokine gene expression *in vivo*. Ten days after transplantation, islet allografts with lymphocyte were surgically obtained from kidneys and the cells were subjected to a quantitative RT-PCR. IL-2 and IFNγ gene expression was strongly suppressed by treatment with RCAN-11R (Fig. 4c,d).

### The effect of RCAN-11R on insulin secretion

**Figure 5:**
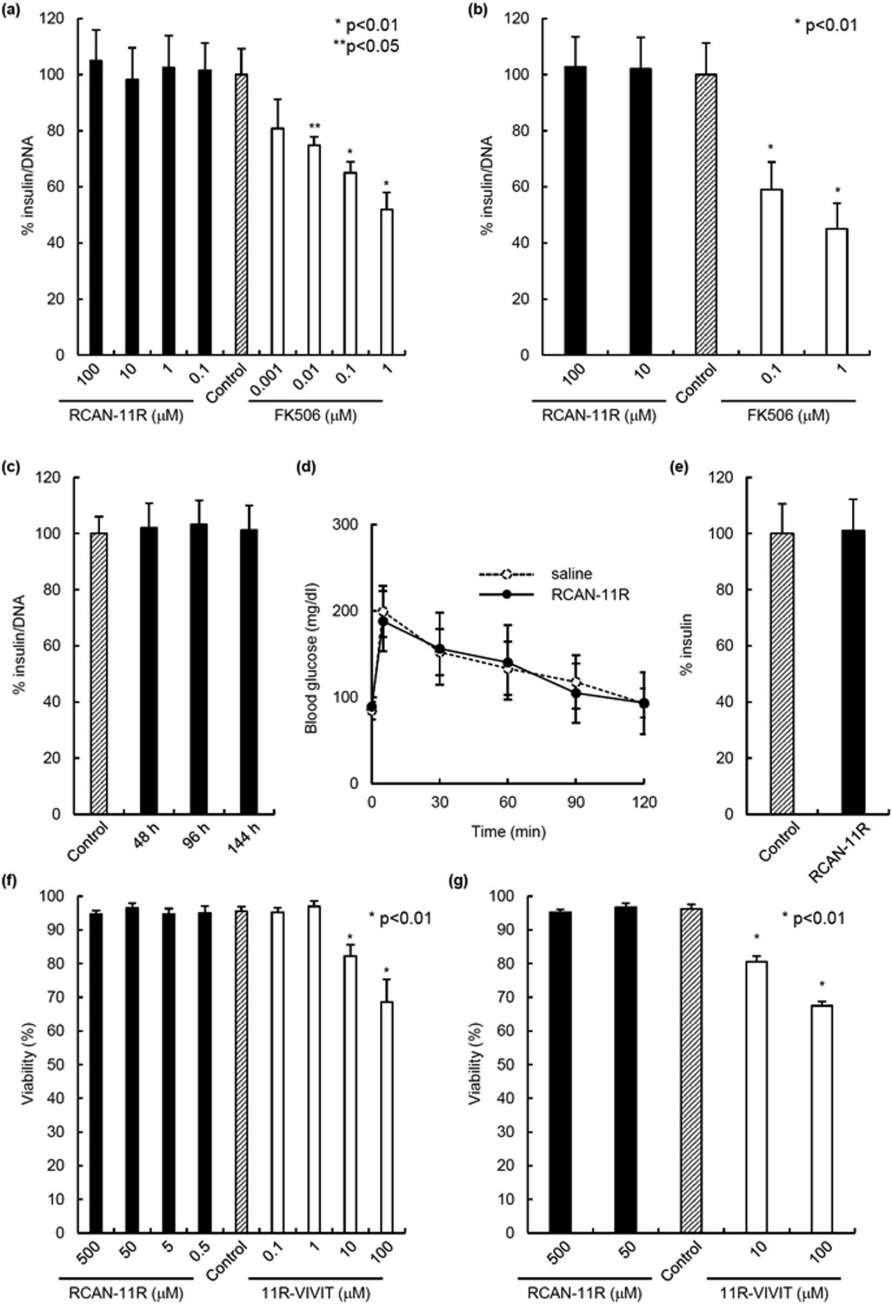
The effects of RCAN-11R on insulin secretion and cell viability. (**a**) Insulin secretion in β-cell line, βTC6 cells. βTC6 cells were added to 96-well plates (50,000 cells/well) with FK506 or with RCAN-11R in complete medium. The medium with FK506 or with RCAN-11R was changed every 24 hours. After 96 hours, the medium was changed and the samples for investigating the release of insulin were taken 1 hour later. The values represent the mean ± SE of five independent experiments. (**b**) Insulin secretion in isolated islets from BALB/c mice. Isolated islets from BALB/c mice were added to 24-well plates (250 IE) with FK506 or with RCAN-11R in complete medium. The medium with FK506 or with RCAN-11R was changed every 24 hours. After 96 hours, the medium was changed and the samples for investigating the release of insulin were taken 1 hour later. The values represent the mean ± SE of five independent experiments. (**c**) Insulin secretion in βTC6 cells with RCAN-11R treatment for 48, 96, or 144 hours. βTC6 cells were added to 96-well plates (50,000 cells/well) with RCAN-11R for 48, 96, or 144 hours and then the medium was changed and the samples for investigating the release of insulin were taken 1 hour later. The values represent the mean ± SE of five independent experiments. (**d**) IPGTT. IPGTT was carried out on normal BALB/c mice (n = 5 each) after treatment with 50 mg/kg of RCAN-11R or saline for ten days. Glucose (2.0 g/kg body weight) was intraperitoneally injected. (**e**) Blood insulin level in mice after RCAN-11R treatment. Blood insulin level in mice after RCAN-11R treatment was evaluated ten days after treatment with 50 mg/kg of RCAN-11R or saline. The mice were fasted overnight and the blood samples were assayed with a mouse insulin ELISA kit. (**f**) Cell viability in β-cell line, βTC6 cells. The cell viability after treatment with RCAN-11R or 11R-VIVIT for 24 h was assessed using FDA/PI staining to visualize the living and dead cells simultaneously. Ten sets of one hundred βTC6 cells (total 1,000 cells) were inspected, and their individual viabilities were visually determined. The average viability was then calculated. *p < 0.01/**p < 0.05 in comparison to control (No treatment). (**g**) Cell viability in isolated islets from BALB/c mice. The cell viability after treatment with RCAN-11R or 11R-VIVIT for 24 h was assessed using FDA/PI staining to visualize the living and dead cells simultaneously. Fifty islets were inspected, and their individual viabilities were visually determined. The average viability was then calculated. *p < 0.01 in comparison to control (No treatment). The values represent the mean ± SE.

We investigated the effect of RCAN-11R on the insulin secretion in βTC6 cells or isolated islets from BALB/c mice after incubation with RCAN-11R or FK506 for 97 h. The insulin secretion did not change at any concentration of RCAN-11R while 0.01 μM FK506 inhibited insulin secretion; the secretion was significantly inhibited (p < 0.05 for 0.01 μM; p < 0.01 for 0.1 μM and 1 μM) in a dose-dependent manner (βTC6 cells; Fig. 5a, islets; Fig. 5b). We also investigated the effect of RCAN-11R on the insulin secretion in βTC6 cells after incubation with RCAN-11R for 48, 96, or 144 h. The insulin secretion did not change at any time (Fig. 5c). These data suggest that RCAN-11R is less toxic than calcineurin inhibitors with regard to insulin secretion.

IPGTT was carried out on normal BALB/c mice after treatment with 50 mg/kg of RCAN-11R or saline for 10 days. The mice were fasted overnight, after which glucose (2.0 g/kg body weight) was injected intraperitoneally. The blood glucose levels of the mice that were treated with RCAN-11R were similar to those of the mice that were treated with saline (Fig. 5d). RCAN-11R treatment did not affect the blood insulin level in mice (Fig. 5e). These data suggest that treatment with 50 mg/kg of RCAN-11R might not affect the secretion of insulin *in vivo*.

### The effect of RCAN-11R on cell viability

Since >10 μM of 11R-VIVIT was found to unexpectedly affect cell viability and this was due to the VIVIT sequence, not the 11R sequence (Supplemental Fig. 3), the cell viability in βTC6 cells or isolated islets from BALB/c mice after treatment with RCAN-11R or 11R-VIVIT was assessed using FDA/PI staining to visualize the living and dead cells simultaneously. RCAN-11R did not affect cell viability at concentrations of 0.5–500 μM, while >10 μM 11R-VIVIT significantly decreased cell viability in a dose-dependent manner (p < 0.01) (βTC6 cells; Fig. 5f, islets; Fig. 5g). These data suggest that RCAN-11R is less toxic than 11R-VIVIT with regard to cell viability.

## Discussion

In the present study, we demonstrated that the RCAN-11R peptide provides immunosuppression for fully mismatched islet allografts in mice by inhibiting calcineurin-NFAT signaling. The Ca^2+^-activated phosphatase calcineurin dephosphorylates NFAT proteins and promotes their nuclear translocation and activation^1, 2, 8^. Calcineurin docks at a site in the conserved NFAT regulatory domain that has the consensus sequence PxIxIT^21^. Although cyclosporine A and FK506 inhibit calcineurin activity, the inhibition of calcineurin outside the immune system has a number of side effects. We previously developed 11R-VIVIT, which selectively interferes with the calcineurin-NFAT interaction without affecting the calcineurin phosphatase activity^7^. VIVIT peptide was developed based on the conserved calcineurin docking site of the NFAT family^8^. However, 11R-VIVIT affected cell viability when used higher concentrations (as shown in Fig. 5) because of the VIVIT sequence. We therefore sought to develop another safer NFAT inhibitor that did not affect cell viability. RCAN is an endogenous calcineurin inhibitor that physically interacts with calcineurin and modulates its phosphatase activity^17–20^. RCAN1 has two different and independent calcineurin-interacting motifs: PxIxxT and the calcineurin inhibitor of calcipressin (CIC). In contrast, the interaction between RCAN3 and Cn appears to be driven solely by the CIC motif. Although RCAN proteins can contain both of these calcineurin binding sites, only the RCAN CIC motif is responsible for inhibiting calcineurin-NFAT-dependent signaling in human T cells^22, 23^. Moreover, it has been crystallographically predicted that the PSVVVH sequence in the RCAN peptide would interact in a similar fashion to the VIVIT peptide^24^. As shown in Fig. 5a,b and c, RCAN-11R did not affect the secretion of insulin *in vitro*, similar to 11R-VIVIT. As shown in Fig. 5d and e, the administration of RCAN-11R was not likely to result in any significant alteration of the disappearance of glucose *in vivo*. Furthermore, the peptide did not affect cell viability as shown in Fig. 5f and g. These data suggest that RCAN-11R may be less toxic than FK506 or 11R-VIVIT.

During the past 20 years, protein transduction domains (PTDs), which are also known as cell penetrating peptides (CPPs), have been characterized for their ability to translocate into live cells^9–13^. PTDs facilitate the transduction of cargo across the membrane, and thus allow the proteins to accumulate within the cell. Although it is likely that PTDs are not all internalized by a single mechanism, one of the principal mechanisms of protein transduction is via electrostatic interaction with the plasma membrane, the subsequent penetration into the cells by macropinocytosis, and the release into the cytoplasm and nuclei by retrograde transport^13, 25–27^. Poly-arginine (polyR) has been shown to exhibit greater efficiency than other PTDs in terms of the delivery of a number of peptides and proteins^9–11, 28^. Thus, we used 11R-PTD in this study.

In conclusion, we developed an immunosuppressive agent, RCAN-11R, which selectively interfered with the calcineurin-NFAT interaction. The novel NFAT inhibitory peptide could be useful as an immunosuppressive agent that is less toxic than calcineurin inhibitors or 11R-VIVIT.

## Materials and Methods

### Peptide synthesis

Peptides (RCAN-11R: KYELHAATDTTPSVVVHVCES-GG-RRRRRRRRRRR; scrambled (sc) RCAN-11R: SAVTHKLESVDPATVYCETHV-GG-RRRRRRRRRRR; 11 R: RRRRRRRRRRR; 11R-VIVIT: RRRRRRRRRRR-GG-MAGPHPVIVITGPHEE; TAT: GRKKRRQRRRPPQ; TAT-VIVIT: GRKKRRQRRRPPQ-GG-MAGPHPVIVITGPHEE) and FITC-conjugated peptides were synthesized by BIOSYNTHESIS (Lewisville, TX, USA). The peptides were purified by preparative reversed-phase HPLC and were >95% pure, with the expected amino acid composition and mass spectra.

### The transduction of the RCAN-11R peptide

Human T lymphocytes (Jurkat, American Type Culture Collection (ATCC), Manassas, VA, USA) were grown in RPMI-1640 culture medium (Sigma-Aldrich, St. Louis, MO, USA) containing 10% fetal bovine serum (FBS, Thermo Scientific HyClone, Utah, USA) and penicillin-streptomycin (50 IU/ml and 50 μg/ml, respectively, Wako Pure Chemical Industries Ltd, Osaka, Japan). The cells were incubated with 10 μM FITC-conjugated RCAN-11R and examined using an Olympus confocal microscope.

### The inhibition of GFP-NFAT-1 nuclear translocation

HEK293 cells (ATCC) were plated onto glass cover slips in 35-mm culture dishes. GFP-NFAT-1 plasmid^6^ was transfected with Lipofectamine 2000 (ThermoFisher Scientific K.K., Yokohama, Japan). After 24 h, the cells were incubated for 2 h with 1 μM FK506 (Sigma-Aldrich) or 20 μM RCAN-11R/scRCAN-11R, after which 500 nM ionomycin (Sigma-Aldrich) was added to the culture medium. The nuclear fluorescence intensity was quantified using a Fluoview device (Olympus, Tokyo, Japan) and measured with AquaCosmos software (Hamamatsu Photonics). The fluorescent intensity of 10 cells was counted; five sets of experiments were conducted. Background fluorescent intensity was subtracted from all experiments.

### Western Blot

Jurkat cells (1 × 10^5^) were treated with 0.1, 1, or 10 μM RCAN-11R for 1 h and with 20 nM PMA (Sigma-Aldrich) and 300 nM ionomycin for an additional 15 min. Whole-cell extracts were fractionated by 10% SDS-PAGE and transferred to reinforced cellulose nitrate membrane. After blocking, the membranes were incubated overnight at 4 °C in TBS buffer (50 mmol/l Tris-HCl, 150 mmol/l NaCl) containing a 1:10,000 dilution of rabbit anti-NFAT1 antibody (Cell Signaling, Danvers, MA, USA), and then incubated for 1 h at room temperature in TBS containing a 1:2,000 dilution of rabbit antibody to IgG coupled to horseradish peroxidase (Cell Signaling). Immunoreactive bands were visualized by incubation with LumiGLO (Cell Signaling) and exposed to light-sensitive film.

### Reporter assay

Jurkat cells (1 × 10^5^) were electroporated with 5 μg of pNFAT-SEAP or pNF-κB-SEAP (Takara Bio, Shiga, Japan) using a GenePulser Xcell apparatus (Bio-Rad, Tokyo, Japan) by a preinstalled procedure. The cells with pNFAT-SEAP or pNF-κB-SEAP were treated with 1 μM FK506 or 20 μM RCAN-11R/scRCAN-11R for 1 h and with 200 nM PMA and 4 μM ionomycin for an additional 12 h. The supernatant was transferred into a new tube and mixed with a Great EscAPe Chemiluminescence Detection Kit (Takara Bio) in cuvettes for luminometry.

### The inhibition of the transcription of interleukin-2 (IL-2)

Jurkat cells (1 × 10^5^) were treated with medium containing peptides or FK506, then incubated with the same amount of medium containing 200 nM PMA and 4 μM ionomycin for an additional 12 h. Total RNA was extracted from cells using the RNeasy Mini Kit (Qiagen, Valencia, CA, USA). After quantifying the RNA by spectrophotometry, 2.5 μg of RNA was heated at 85 °C for 3 min and then reverse transcribed into cDNA in a 25 μl solution containing 200 U of Superscript II RNase H-RT (Thermo Fisher Scientific, Yokohama, Japan), 50 ng random hexamers (Thermo Fisher Scientific), 160 μmol/l dNTP and 10 nmol/l dithiothreitol. The reaction consisted of 10 min at 25 °C, 60 min at 42 °C and 10 min at 95 °C. Polymerization reactions were performed as shown in the DNA purification and PCR section. Quantification of the mRNA levels was carried out using the TaqMan real-time PCR system, according to the manufacturer’s instructions (Applied Biosystems, Foster City, CA, USA). PCR was performed for 40 cycles, including 2 min at 50 °C and 10 min at 95 °C as initial steps. In each cycle, denaturation was performed for 15 s at 95 °C and annealing/extension was performed for 1 min at 60 °C. PCR was carried out in 20 μl of solution using cDNAs synthesized from 1.11 ng of total RNA. For each sample, the expression of mRNA was normalized by dividing the β-actin expression level. Primers for mouse IL-2, IFNγ, and β-actin are commercially available (Assays-on-Demand Gene Expression Products; Applied Biosystems).

### The inhibition of the production of IL-2

Jurkat cells (1 × 10^5^) were treated with medium containing peptides or FK506, then incubated with the same amount of medium containing 200 nM PMA (Sigma-Aldrich) and 4 μM ionomycin for an additional 12 h. IL-2 protein was detected in the supernatant of cells using an ELISA kit according to the manufacturer’s instructions (eBioscience, San Diego, CA, USA).

### The effects of RCAN-11R on islet transplantation

All of the mouse studies were approved by the review committee of the University of the Ryukyus. C57BL/6 mice were used as donors and BALB/c mice were used as recipients. The recipients were rendered diabetic by a single intraperitoneal injection of 220 mg/kg of streptozotocin (STZ, Sigma-Aldrich). Hyperglycemia was defined when a glucose level of >350 mg/dl was detected twice, consecutively, after STZ injection. The islets were isolated, and 500 freshly isolated islets were transplanted into the renal subcapsular space of the left kidney of the diabetic mice, as previously described^7, 29^. During the 50-day post-transplantation period, the non-fasting blood glucose levels were monitored every day. Graft failure was determined when the non-fasting blood glucose level exceeded 200 mg/dl for two consecutive days. Glucose was measured using an ACCU-CHEK^®^ Compact Plus (Roche Diagnostics K. K., Tokyo, Japan) according to the manufacturer’s instructions.

### Intraperitoneal glucose tolerance testing

Intra-peritoneal glucose tolerance testing (IPGTT) was performed ten days after transplantation or after treatment with 50 mg/kg of RCAN-11R or saline. The mice were fasted overnight, after which glucose (2.0 g/kg body weight) was injected intraperitoneally. The blood glucose levels were measured before injection and at 5, 30, 60, 90, and 120 minutes after injection. No statistical differences in either the pre-transplantation blood glucose levels or the pre-transplantation body weight were observed between the two groups of mice.

### Cytokine gene expression *in vivo*

Ten days after transplantation, islet allografts with lymphocyte were surgically obtained from kidneys. Total RNA was isolated by RNeasy kit (Qiagen), and RNA concentration was measured spectrophotochemically. Total RNA (1 μg) was subjected to first-strand cDNA synthesis and amplified by and the cells were subjected to a quantitative RT-PCR.

### The effects of RCAN-11R on insulin secretion

βTC6 cells (ATCC) were grown in RPMI-1640 culture medium containing 10% FBS and 11.1 mM glucose. βTC6 cells were added to 96-well plates (50,000 cells/well) with FK506 or with RCAN-11R in complete medium. Isolated islets from BALB/c mice were added to 24-well plates (250 islet equivalents; IE) with FK506 or with RCAN-11R in complete medium. The medium with FK506 or with RCAN-11R was changed every 24 hours. After 48, 96, or 144 hours, fresh medium was added; the samples taken to investigate the release of insulin were taken 1 hour later and assayed with a mouse insulin ELISA kit (Shibayagi, Shibukawa, Gunma, Japan).

Blood insulin level in mice after RCAN-11R treatment was evaluated ten days after treatment with 50 mg/kg of RCAN-11R or saline. The mice were fasted overnight and the blood samples were assayed with a mouse insulin ELISA kit.

### Cell viability

Cell viability was assessed after treatment with RCAN-11R, 11R, 11R-VIVIT, TAT, or TAT-VIVIT for 24 h using double fluorescein diacetate/propidium iodide (FDA/PI, Sigma-Aldrich) staining to visualize the living and dead cells simultaneously^30–32^. Ten sets of 100 βTC6 cells (total 1,000 cells) or fifty islets from BALB/c mice were inspected, and their individual viabilities were visually determined. Finally, their average viability was calculated^33^.

### Statistical Analyses

The values for the data are presented as the means ± SE. To compare the data among the groups, a repeated measures ANOVA or the Kaplan-Meier log-rank test was used. The differences between the groups were considered to be significant for values of P < 0.05.

All methods were performed in accordance with the relevant guidelines and regulations.

## Electronic supplementary material

Supplementary Information
