# Supplementary material for: RCAN-11R peptide provides immunosuppression for fully mismatched islet allografts in mice
Source: Sci Rep. 2017 Jun 8;7:3043. doi: 10.1038/s41598-017-02934-3 (PMC5465209; doi:10.1038/s41598-017-02934-3)
Supplement: Supplementary file 1 — Supplementary Information [file 41598_2017_2934_MOESM1_ESM.doc]

**Original Article**

RCAN-11R peptide provides immunosuppression for fully mismatched islet allografts in mice.

Hirofumi Noguchi ^1,*^, Koji Sugimoto ^2^, Chika Miyagi-Shiohira ^1^, Yoshiki Nakashima ^1^, Naoya Kobayashi ^3^, Issei Saitoh ^4^, Masami Watanabe ^5^, Yasufumi Noguchi ^6^

1. Department of Regenerative Medicine, Graduate School of Medicine, University of the Ryukyus, Okinawa 903-0215, Japan.

2. Department of Surgery, Yoshinogawa Medical Center, Tokushima 776-8511, Japan

3. Okayama Saidaiji Hospital, Okayama 704-8192, Japan.

4. Division of Pediatric Dentistry, Graduate School of Medical and Dental Science, Niigata University, Niigata 951-8514, Japan.

5. Department of Urology, Okayama University Graduate School of Medicine, Dentistry and Pharmaceutical Sciences, Okayama 700-8558, Japan.

6. Department of Socio-environmental Design, Hiroshima International University, Hiroshima 737-0112, Japan.

There was no Competing Financial Interests.


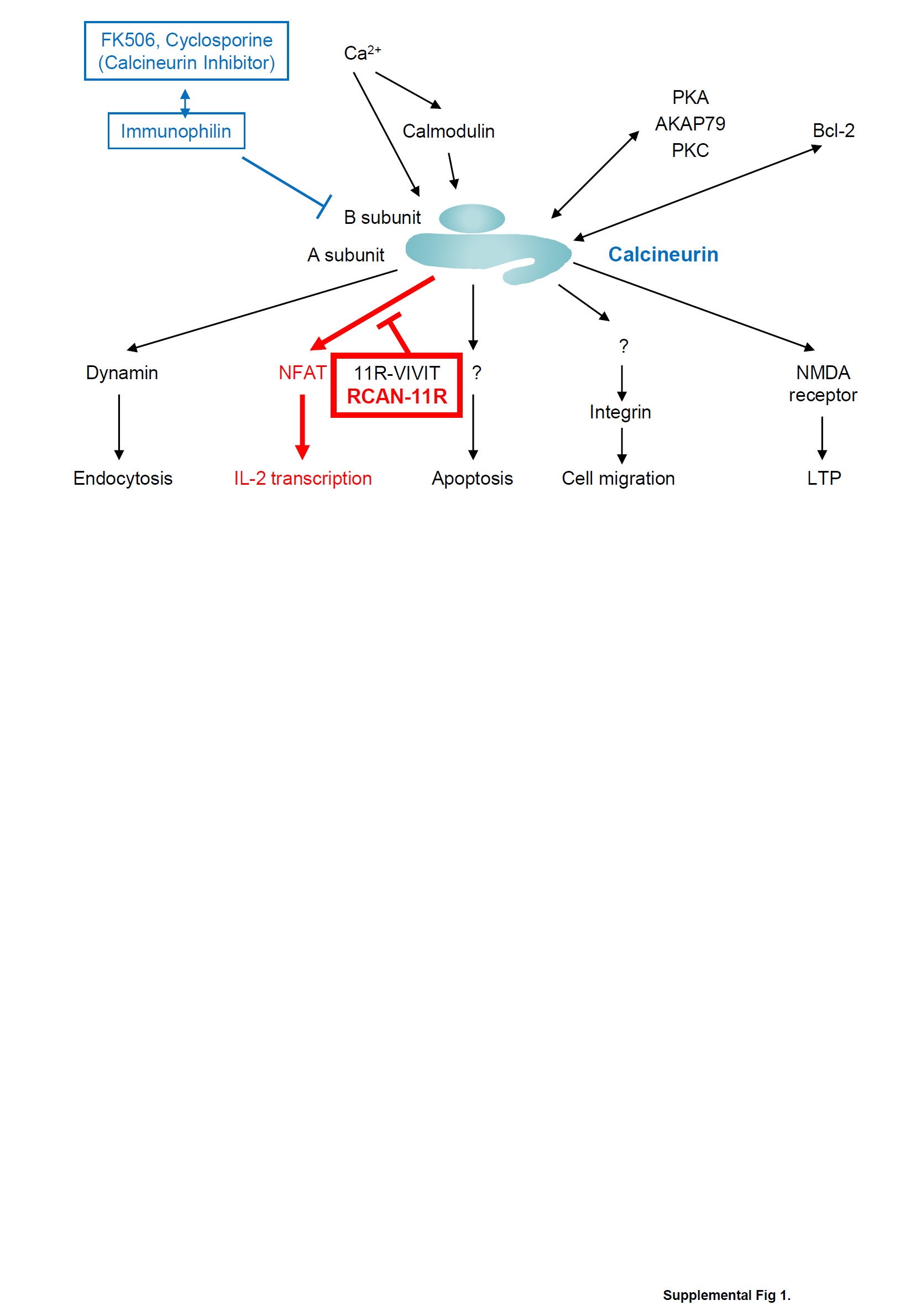


**SUPPLEMENTAL FIGURE LEGENDS**

**Supplemental Figure 1: Model of the inhibition of calcineurin-NFAT signaling by RCAN-11R and 11R-VIVIT.**

RCAN is responsible for the inhibition of calcineurin-NFAT signaling. Similarly to the VIVIT peptide, the RCAN-derived peptide selectively interferes with the calcineurin-NFAT interaction without affecting the calcineurin phosphatase activity. RCAN-derived peptide as a N-terminal fusion protein with 11R (RCAN-11R) were synthesized to facilitate the efficient *in vivo* delivery of the NFAT inhibitor peptide into T cells.

**Supplemental Figure 2: The effects of RCAN1-11R, RCNA2-11R, and RCAN3-11R on IL2 mRNA expression.**

**(A)** The sequence of RCAN1-11R, RCNA2-11R, and RCAN3-11R. **(B)** The inhibition of IL-2 transcription. Jurkat cells were treated with 20 μM RCAN1-11R, RCNA2-11R, and RCAN3-11R for 1h, then with 200 nM PMA and 4 μM ionomycin for an additional 12h. The cells were subjected to a quantitative RT-PCR. The values represent the mean ± SE.

**Supplemental Figure 3: The effects of 11R, 11R-VIVIT, TAT, and TAT-VIVIT on cell viability.**

**(A)** The sequence of 11R, 11R-VIVIT, TAT and TAT-VIVIT. **(B)** Cell viability in β-cell line, βTC6 cells. The cell viability after treatment with 100μM of 11R, 11R-VIVIT, TAT and TAT-VIVIT for 24 h was assessed using FDA/PI staining to visualize the living and dead cells simultaneously. Ten sets of one hundred βTC6 cells (total 1,000 cells) were inspected, and their individual viabilities were visually determined. The average viability was then calculated. *p<0.01 in comparison to control (No treatment). The values represent the mean ± SE.
